# Trans‐right ventricle metabolite gradients in obesity highlight multiple metabolic pathways

**DOI:** 10.14814/phy2.70323

**Published:** 2025-05-11

**Authors:** Hanna M. Knauss, Mythri Ambatipudi, Leah B. Kosyakovsky, Matthew S. Herzig, Jessica K. Wang, Elizabeth E. Liu, Emily S. Lau, Jenna N. McNeill, Xu Shi, Robert E. Gerszten, Naomi M. Hamburg, Gregory D. Lewis, Jeremy M. Robbins, Jennifer E. Ho

**Affiliations:** ^1^ Division of Cardiology Beth Israel Deaconess Medical Center Boston Massachusetts USA; ^2^ Division of Cardiology Massachusetts General Hospital Boston Massachusetts USA; ^3^ Division of Pulmonary and Critical Care Medicine Duke University Hospital Durham North Carolina USA; ^4^ Whitaker Cardiovascular Institute, School of Medicine Boston University Boston Massachusetts USA

**Keywords:** metabolite gradients, obesity, pulmonary hypertension, trans‐right ventricle

## Abstract

Obesity and metabolic dysfunction are associated with pulmonary vascular remodeling, yet molecular mechanisms remain poorly understood. We sought to study trans‐right ventricular (RV) metabolite gradients to elucidate potential molecular pathways operant among individuals with obesity and pulmonary hypertension. In this study, 38 individuals with obesity (mean age 58 years, 68% women, average BMI 36.6 kg/m^2^) underwent invasive right heart catheterization. Multi‐site blood sampling from the superior vena cava and pulmonary artery was performed to assess trans‐RV gradients, with targeted metabolite profiling using liquid chromatography‐mass spectrometry. We found 56 metabolites with significant trans‐RV gradients (FDR *q* < 0.05), including intermediates of fatty acid oxidation, the tricarboxylic acid cycle, and nucleotide metabolism. Further, trans‐RV gradients in lipid and purine metabolism were associated with BMI and related cardiometabolic traits, such as waist circumference, insulin resistance, and serum lipids. Finally, differential levels of bile acids, intermediates of lipid peroxidation, and nucleotide metabolism across the RV were associated with pulmonary hypertension. In conclusion, trans‐RV metabolite gradients among individuals with obesity reveal alterations in metabolites representative of molecular pathways such as fatty acid oxidation, and others correlated with cardiometabolic traits and/or pulmonary hypertension, including orotic acid, bile acids, and acylcarnitines.

## 1 INTRODUCTION

Obesity is increasingly recognized as a heterogeneous disease state associated with diverse metabolic phenotypes and a range of cardiovascular manifestations (Ho et al., 2016). Recent studies have shown that obesity is associated with pulmonary hypertension (PH) across all hemodynamic subtypes (Frank et al., 2020). Human studies highlight potential contributions from dyslipidemia and insulin resistance, and experimental PH models substantiate metabolic dysregulation leading to endothelial dysfunction and pulmonary vascular remodeling, though the exact underlying mechanisms remain poorly understood (Brittain et al., 2017; Hemnes et al., 2019).

Metabolite profiling has been utilized to understand specific molecular pathways underlying cardiometabolic disease and stratify individuals with obesity who are at higher risk for metabolic comorbidities (Beyene et al., 2023; Ho et al., 2016). Large‐scale molecular profiling has also been used in prior studies that leveraged multi‐site blood sampling to infer uptake or release of metabolites across specific organs (Lewis et al., 2010). For instance, concurrent sampling of the superior vena cava (SVC) and pulmonary artery (PA) has been performed to better understand potential mechanisms of selected individuals with PH (Chouvarine et al., 2020; Lewis et al., 2016). Prior studies in cardiometabolic disease and PH support alterations in energy utilization across the liver, visceral adipose tissue, intestines, and skeletal muscle (Kelley, 2005; Koves et al., 2008). Since these sites drain blood through tributaries that ultimately terminate in the IVC, differences in metabolite profiles between the SVC and PA (trans‐RV metabolite gradients) may reflect important metabolic signatures evident in the splanchnic and hepatic circulation that in turn get released into the pulmonary circulation. Alternatively, differences between metabolite concentrations in the SVC and PA may be attributed to energy utilization across the RV. While prior studies have focused predominantly on patients with pulmonary arterial hypertension, no study has examined trans‐RV metabolite profiling in obesity and obesity‐associated PH.

In this study, we leveraged large‐scale metabolite profiling coupled with multi‐site sampling across the SVC and PA to examine trans‐RV metabolite gradients among individuals with obesity. We hypothesized that metabolic pathways displaying significant trans‐RV metabolite gradients would inform our understanding of the molecular mechanisms of obesity‐associated PH.

## 2 METHODS

### 2.1 Participants

Study participants were previously enrolled in the Cardiometabolic disease and pulmonary hypertension (METCPET) study, as described previously (Kosyakovsky et al., 2024). In brief, the METCPET study was a prospective investigation that evaluated the association between metabolic disease and pulmonary hypertension. Participants were required to be 30–80 years of age with a history of body mass index (BMI) ≥30 kg/m^2^ with exertional dyspnea (Modified Medical Research Council Scale grade ≥1). Individuals with known cardiovascular disease, systolic heart failure, chronic kidney disease (estimated GFR <45 mL/min/1.73m^2^), severe liver disease, anemia (Hb <9 mg/dL), primary pulmonary arterial hypertension (PAH), and/or moderate to severe chronic obstructive pulmonary disease (COPD) were excluded from the study. Forty participants underwent right heart catheterization (RHC). Of the 40 participants, 2 were missing baseline samples from the SVC and/or PA and were excluded from our analysis. All participants provided written informed consent and the study protocol was approved by the appropriate institutional review boards, including the Committee on Clinical Investigation at Beth Israel Deaconess Medical Center and the Partners Human Research Committee at Massachusetts General Hospital.

### 2.2 Clinical assessment

Detailed medical histories and physical examinations were obtained from all study participants. Fasting plasma samples, BMI, and waist circumference were collected at the initial study visit. Laboratory assessment included serum low‐density lipoprotein (LDL) and glycated hemoglobin (A1c). The homeostatic model of insulin resistance (HOMA‐IR) was calculated as [fasting insulin (μLU/mL) × fasting glucose (mmol/mL)/22.5] (Matthews et al., 1985).

### 2.3 Right heart catheterization

Following an overnight fast, participants underwent insertion of a pulmonary artery catheter via the internal jugular vein. Resting supine hemodynamic measures were obtained, including cardiac output (CO), mean pulmonary artery pressure (mPAP), pulmonary capillary wedge pressure (PCWP), and pulmonary vascular resistance (PVR). Cardiac output was assessed by the direct Fick method. Pulmonary hypertension was defined as a mean PA pressure >20 mm Hg and further classified as pre‐capillary, post‐capillary, combined pre‐ and post‐capillary, and unclassified PH according to standard definitions (Humbert et al., 2022).

### 2.4 Plasma metabolite profiling and measurements

Fasting blood sampling at rest was performed from the SVC and PA ports from the indwelling pulmonary artery catheter, which were immediately processed and frozen at −80°C until assayed. Targeted metabolite profiling was executed as previously described (Benson et al., 2023). Amino acids, lipids, and other polar metabolites were measured using a hydrophilic interaction liquid chromatography (HILIC) column (Waters; Milford, MA) coupled to an Orbitrap mass spectrometer (Thermo Fisher Scientific; Waltham, MA). Analyses were carried out using electrospray ionization in the positive mode. Sugars, purines, and other intermediary metabolites were separated using amide chromatography (Waters Xbridge Amide column) and measured using target negative ion mode coupled to an Agilent 6490 triple quadrupole mass spectrometer (Agilent; Santa Clara, CA). Isotope‐labeled internal standards were monitored in each sample, and pooled plasma samples were used to assess quality control and reproducibility. We used a linear scaling approach to the nearest pooled plasma sample. Median inter‐assay coefficients of variation (CV) of samples in the amide and HILIC columns were 4.9% and 2.6%, respectively. For metabolites dual‐profiled under amide and HILIC modes of liquid chromatography, the value with the lower inter‐assay CV was selected.

### 2.5 Statistical analyses

Baseline clinical and hemodynamic measures were compared for individuals with and without resting PH. Metabolite data were natural log‐transformed due to right‐skewed distributions. Trans‐RV metabolite gradients (∆MET) were then calculated as PA minus SVC concentrations, with “positive” ∆MET representing metabolites with higher concentrations of metabolite in the PA versus SVC; conversely, metabolites with “negative” gradients had higher concentrations in the SVC versus PA. We used paired *t*‐tests to identify significant ∆MET for each metabolite. To account for multiple hypothesis testing, we used a false discovery rate (FDR) *q* < 0.05.

We next examined clinical correlates of ∆MET using partial correlation coefficients adjusted for age and sex after standardizing ∆MET to a mean of 0 and a standard deviation of 1. Clinical correlates of interest included BMI, waist circumference, serum triglyceride levels, HOMA‐IR, and low‐density lipoprotein (LDL). Participants who were missing data for any of these five clinical correlates (*n* = 1 for HOMA‐IR and n = 1 for LDL) were excluded, and participants with pre‐existing diabetes (*n* = 2) were excluded from the HOMA‐IR analysis. To examine differences in ∆MET among individuals with obesity, with and without PH, we used multivariable logistic regression models adjusted for age, sex, and BMI. All analyses were performed in R studio using R version 4.2.1.

## 3 RESULTS

### 3.1 Clinical and hemodynamic characteristics

Clinical and hemodynamic characteristics are outlined in Table S1. A total of 38 participants were included in the study, with a mean age of 58 ± 12 years, 68% female, and an average BMI of 36.6 ± 5.5 kg/m^2^. Among this group, 18 (47.3%) had PH, with 11% meeting criteria for pre‐capillary, 39% post‐capillary, 11% combined, and 39% unclassified PH.

### 3.2 Trans‐right ventricle metabolite gradients

**FIGURE 1:**
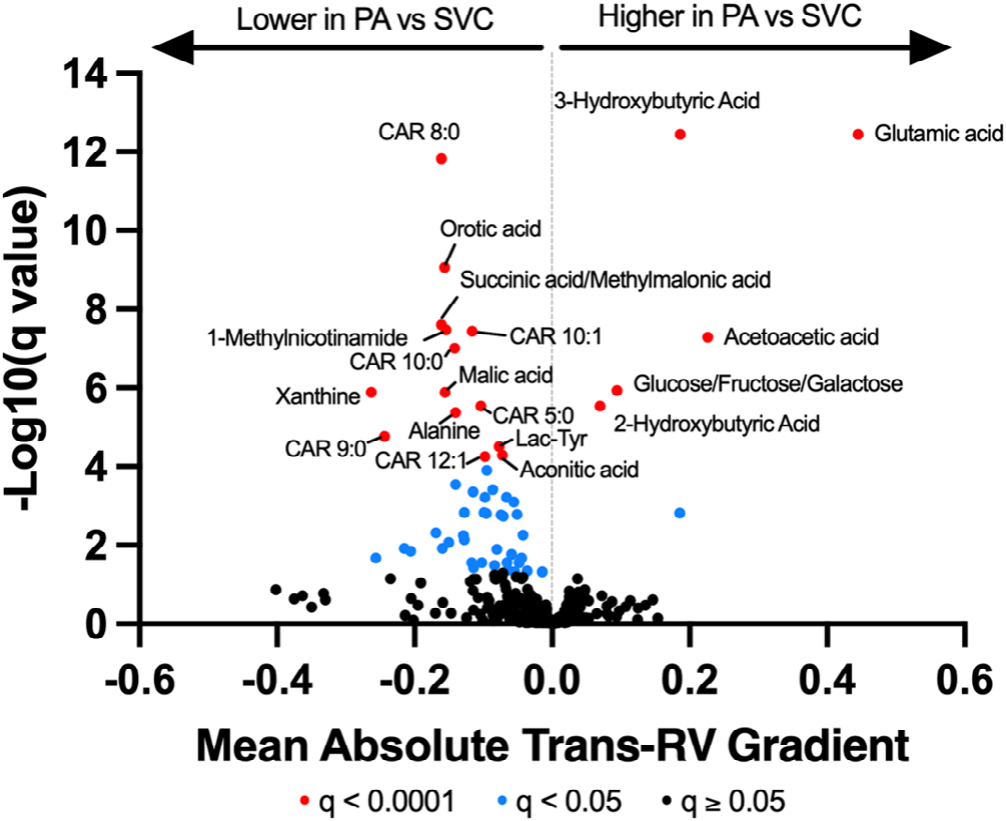
Trans‐right ventricle metabolite gradients in individuals with obesity undergoing right heart catheterization. Trans‐RV gradients of 379 metabolites. *x*‐axis indicates absolute difference between PA versus SVC log‐transformed metabolite concentrations. Red color indicates *q* < 0.0001, blue color indicates 0.0001 < *q* < 0.05.

Of the 379 metabolites studied, 56 metabolites displayed significant trans‐RV gradients (FDR *q* <0.05; Figure 1, Figure S1 and Table S2), with the most significant findings (FDR *q* <0.0001) displayed in Table 1. A total of six of 56 (11%) displayed higher PA versus SVC concentrations, indicating positive gradients across the RV, including intermediates of ketogenesis (acetoacetic acid, 2‐hydroxybutyric acid, 3‐hydroxybutyric acid), monosaccharides (glucose/fructose/galactose), and glutamic acid. For instance, among the top hits, we found that 3‐hydroxybutyric acid had a 0.19‐SD higher PA versus SVC concentration (mean difference 0.19 ± 0.01), glutamic acid was 0.44 SD higher (0.44 ± 0.03), and acetoacetic acid was 0.23‐SD higher (0.23 ± 0.03, FDR *q* <0.0001 for all).

**TABLE 1:** Top trans‐right ventricular metabolite gradients.

Metabolite	PA level	SVC level	Mean difference	SE	*p*‐value	*q*‐value
*Positive Trans‐RV Gradients: Higher in PA* versus *SVC*						
3‐Hydroxybutyric acid	0.20 (0.87)	0.01 (0.86)	0.19	0.01	1.9 × 10^−15^	<0.001
Glutamic acid	0.28 (0.23)	−0.16 (0.33)	0.44	0.03	1.9 × 10^−15^	<0.001
Acetoacetic acid	0.19 (0.54)	−0.04 (0.54)	0.23	0.03	1.1 × 10^−9^	<0.001
Glucose/Fructose/Galactose_waterloss	0.06 (0.11)	−0.04 (0.13)	0.09	0.01	3.1 × 10^−8^	<0.001
2‐Hydroxybutyric acid	0.05 (0.51)	−0.02 (0.51)	0.07	0.01	1.1 × 10^−7^	<0.001
*Negative Trans‐RV Gradients: Lower in PA* versus *SVC*						
CAR 8:0	−0.63 (0.83)	−0.47 (0.83)	−0.16	0.01	1.2 × 10^−14^	<0.001
Orotic acid	−0.23 (0.29)	−0.07 (0.30)	−0.16	0.02	9.1 × 10^−12^	<0.001
Succinic acid/Methylmalonic acid	−0.76 (0.26)	−0.59 (0.24)	−0.16	0.02	3.3 × 10^−10^	<0.001
1‐Methylnicotinamide	−0.54 (0.51)	−0.38 (0.50)	−0.15	0.02	5.3 × 10^−10^	<0.001
CAR 10:1	−0.36 (0.49)	−0.24 (0.48)	−0.12	0.01	6.8 × 10^−10^	<0.001
CAR 10:0	−0.56 (0.77)	−0.42 (0.78)	−0.14	0.02	2.3 × 10^−9^	<0.001
Xanthine	−0.60 (0.35)	−0.34 (0.31)	−0.26	0.04	4.1 × 10^−8^	<0.001
Malic acid	−0.49 (0.39)	−0.33 (0.37)	−0.16	0.02	4.1 × 10^−8^	<0.001
CAR 5:0	−0.26 (0.41)	−0.16 (0.40)	−0.10	0.02	1.0 × 10^−7^	<0.001
Alanine	−0.30 (0.33)	−0.16 (0.30)	−0.14	0.02	1.7 × 10^−7^	<0.001
CAR 9:0	−0.69 (0.77)	−0.44 (0.66)	−0.24	0.04	7.3 × 10^−7^	<0.001
Lac‐Tyr	−0.33 (0.55)	−0.26 (0.55)	−0.08	0.01	1.4 × 10^−6^	<0.001
Aconitic acid	−0.11 (0.27)	−0.03 (0.25)	−0.07	0.01	2.4 × 10^−6^	<0.001
CAR 12:1	−0.32 (0.57)	−0.22 (0.57)	−0.10	0.02	2.8 × 10^−6^	<0.001

*Note*: PA level and SVC level represent mean (SD). Mean difference in standard deviation units of log‐transformed metabolite. The *q*‐value is representative of false discovery rate (FDR)‐adjusted *p*‐value.

By contrast, 50 (89%) metabolites demonstrated negative ∆MET (lower in PA vs. SVC), and included metabolites representing TCA cycle (malic acid, aconitic acid, succinic acid/methylmalonic acid), nucleotide metabolism (orotic acid, xanthine), and prostacyclin activation pathways (1‐methylnicotinamide). The top metabolites displaying lower PA versus SVC concentrations included CAR 8:0 (mean difference −0.16 ± 0.01), orotic acid (−0.16 ± 0.02), succinic acid/methylmalonic acid (−0.16 ± 0.02), 1‐methylnicotinamide (−0.15 ± 0.02), CAR 10:1 (−0.12 ± 0.01), and CAR 10:0 (−0.14 ± 0.02, FDR *q* < 0.0001 for all).

### 3.3 Association of Cardiometabolic Traits with trans‐right ventricle metabolite gradients

We next examined associations of ∆MET with cardiometabolic traits. Of the 379 metabolites profiled, we found 37 (10%) trans‐RV metabolite gradients with suggestive correlations to at least one cardiometabolic trait of interest (*p* < 0.05 for all, including BMI, waist circumference, triglyceride levels, HOMA‐IR, and/or LDL; Table 2), though none met an FDR *q*‐value threshold of <0.05. BMI was correlated with 16 metabolites, 6 of which indicated positive correlations, including intermediates in purine degradation (adenine, allantoin), nicotinamide metabolism (niacinamide), and gluconeogenesis (pyruvic acid). Conversely, BMI was negatively associated with 10 trans‐RV metabolite gradients, including intermediates of arginine metabolism (N‐acetylornithine) and several lipid intermediates such as sphingolipids (C24 1‐deoxyceramide), glycerolipids (DG 34:3, DG 36:2), and triacylglycerols (TG 44:2, TG 46:3).

**TABLE 2:** Correlation of trans‐right ventricle metabolite gradients with BMI and other cardiometabolic traits.

Metabolite	BMI	WC	HOMA‐IR	HbA1c	TG
	*r* (*p*)	*r* (*p*)	*r* (*p*)	*r* (*p*)	*r* (*p*)
Adenine	0.45 (0.01)	0.36 (0.03)	0.19 (0.30)	0.12 (0.50)	0.04 (0.81)
Allantoin	0.38 (0.02)	0.21 (0.22)	0.27 (0.12)	0.28 (0.09)	0.05 (0.76)
C20:4‐Phe	0.48 (0.003)	0.49 (0.002)	0.32 (0.07)	0.32 (0.05)	0.04 (0.83)
C24:1‐Deoxyceramide	−0.44 (0.01)	−0.42 (0.01)	−0.36 (0.04)	−0.18 (0.31)	−0.11 (0.52)
DG 34:3	−0.39 (0.02)	−0.23 (0.18)	−0.02 (0.90)	−0.09 (0.61)	−0.03 (0.85)
DG 36:2	−0.35 (0.04)	−0.21 (0.23)	−0.07 (0.69)	−0.08 (0.65)	−0.10 (0.56)
LPC 19:1	−0.36 (0.03)	−0.22 (0.20)	−0.10 (0.58)	−0.16 (0.36)	−0.13 (0.44)
N‐Acetylornithine	−0.36 (0.03)	−0.22 (0.19)	−0.05 (0.79)	0.03 (0.85)	−0.12 (0.49)
N‐Arachidonoyl Taurine	−0.36 (0.03)	−0.41 (0.01)	−0.34 (0.06)	−0.39 (0.02)	−0.16 (0.35)
Niacinamide	0.53 (<0.01)	0.59 (<0.01)	0.35 (0.048)	0.07 (0.69)	0.08 (0.63)
Pyruvic acid	0.36 (0.03)	0.27 (0.11)	0.52 (<0.01)	0.19 (0.27)	0.34 (0.04)
S‐adenosyl‐L‐homocysteine	−0.44 (0.01)	−0.39 (0.02)	−0.18 (0.31)	0.00 (0.99)	−0.21 (0.23)
Stearoyl.EA.2	0.48 (0.003)	0.43 (0.01)	0.27 (0.13)	0.03 (0.86)	−0.06 (0.74)
TG 44:2	−0.36 (0.03)	−0.39 (0.02)	−0.27 (0.13)	−0.29 (0.09)	−0.11 (0.51)
TG 46:3	−0.4 (0.02)	−0.25 (0.15)	−0.42 (0.01)	−0.14 (0.43)	−0.16 (0.35)
2‐Hydroxyglutaric.acid	−0.36 (0.03)	‐0.19 (0.28)	−0.17 (0.35)	−0.15 (0.39)	−0.10 (0.56)

Abbreviations: A1c, glycated hemoglobin; BMI, body mass index; HOMA‐IR, homeostatic model assessment for insulin resistance; LDL, low‐density lipoprotein; TG, triglyceride levels; *r*, partial correlation coefficient; RV, right ventricle. WC, waist circumference.

We found overlap in the 16 BMI‐associated trans‐RV metabolite gradients with other traits, including eight associated with waist circumference as a measure of abdominal adiposity with consistent directionality, one with triglyceride levels, four with insulin resistance as measured by HOMA‐IR, and one with LDL (heat map displayed in Figure S2, full results in Table S3).

### 3.4 Associations of trans‐right ventricle metabolite gradients with pulmonary hypertension

**FIGURE 2:**
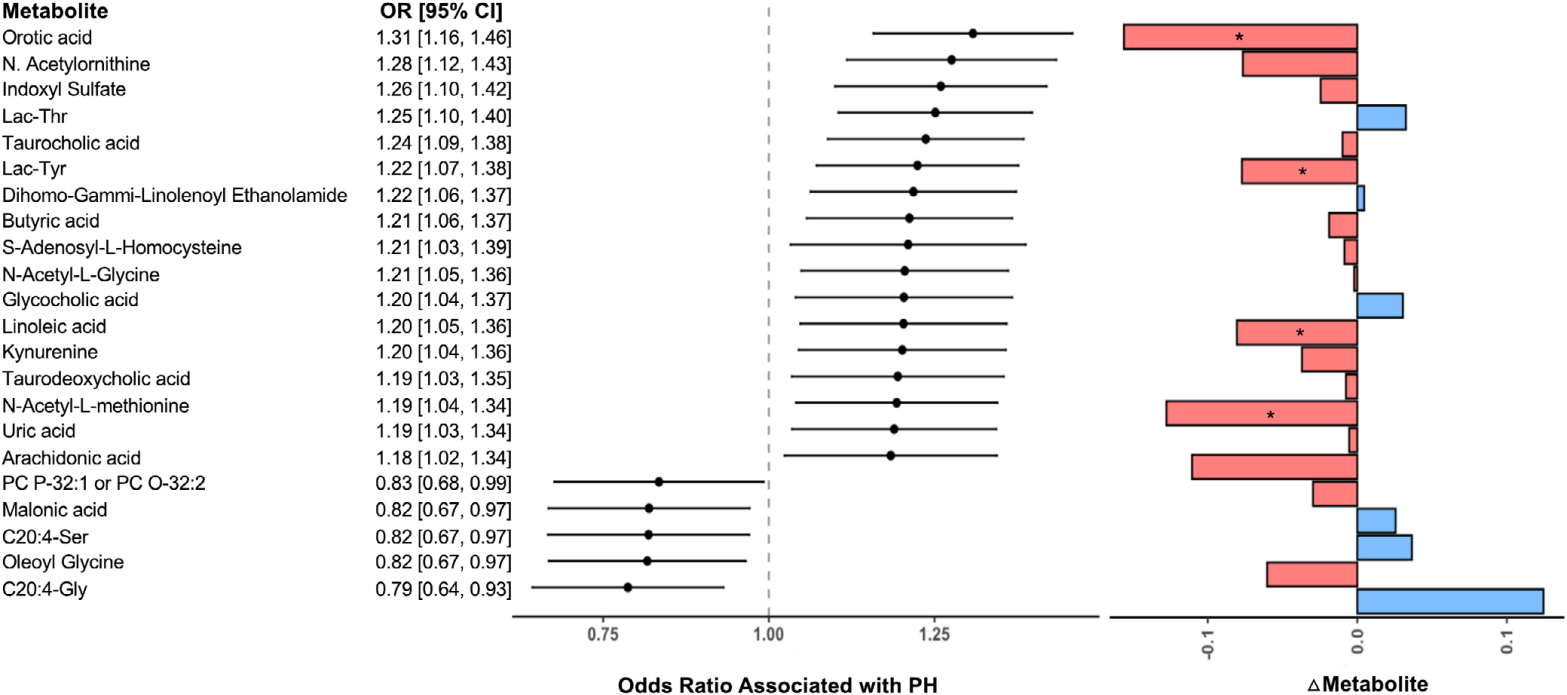
Trans‐RV metabolite gradients significantly associated with pulmonary hypertension. Left: Association of trans‐RV metabolite gradient with odds of pulmonary hypertension in individuals with obesity. Analyses are adjusted for age, sex, and BMI. Right: Mean absolute gradients of 22 metabolites significantly (*p* < 0.05) associated with pulmonary hypertension. *Denotes metabolites with significant (*q* < 0.05) trans‐RV gradients.

Given the known association of cardiometabolic disease with PH, we examined associations of ∆MET with PH as potential underlying contributors. We identified 22 metabolites representative of diverse cellular pathways with trans‐RV gradients associated with PH. Of these 22 metabolite gradients, 17 were associated with higher odds, and 5 with lower odds of PH (Figure 2). Metabolites with ∆MET associated with a higher likelihood of PH included intermediates of tryptophan metabolism (kynurenine, indoxyl sulfate), bile acids (taurodeoxycholic acid, taurocholic acid, glycocholic acid), lipid peroxidation (arachidonic acid, linoleic acid), and nucleotide metabolism (orotic acid, uric acid). Orotic acid's trans RV gradient demonstrated the strongest relationship with PH: orotic acid was associated with a 31% greater odds of PH (OR 1.31, 95% CI 1.16–1.46, *p* = 0.001). Similarly, trans‐RV N‐acetylornithine, lac‐thr, and indoxyl sulfate were also associated with greater odds of PH (OR 1.25–1.28, *p* < 0.05). Further, lac‐tyr, linoleic acid, N‐acetyl‐L‐methionine, and orotic acid all displayed significant trans‐RV gradients (OR 1.19–1.31, *p* < 0.05). Associations of all 379 metabolite gradients with PH are shown in Table S4.

By contrast, metabolites with ∆MET associated with a lower likelihood of PH included primarily lipid intermediates, such as C20:4‐Gly (OR 0.79, 95% CI 0.64–0.93, *p* = 0.003), C20.4. Ser (OR 0.82, 95% CI 0.67–0.97, *p* = 0.01), malonic acid (OR 0.82, 95% CI 0.67–0.97, *p* = 0.01), and oleoyl (OR 0.82, 95% CI 0.67–0.97, *p* = 0.01). There were no metabolite gradients correlating with lower odds of PH that demonstrated significant trans‐RV gradients.

## 4 DISCUSSION

We measured targeted plasma metabolites simultaneously from SVC and PA samples from individuals with obesity and related ∆MET to cardiometabolic traits and PH status. Our findings are three‐fold: First, we highlight the differential concentration of key metabolites across the RV, including metabolites previously implicated in the metabolism of ketones, branched‐chain amino acids, the citric acid cycle, and nucleotides. Differences in metabolite concentrations between the SVC and PA may suggest contributions from hepatic and splanchnic circulation, or conversely may represent increased consumption across the RV. Second, multiple significant trans‐RV metabolite gradients, including intermediates of lipid and purine metabolism, were associated with BMI and displayed overlap with other cardiometabolic traits. Finally, we identified trans‐RV metabolite gradients associated with PH, including intermediates of tryptophan degradation, nucleotide metabolism, fatty acid synthesis, and bile acids. Taken together, our findings highlight potential molecular pathways operant in obesity‐associated PH, implicating metabolic pathways underlying both conditions.

Recent studies have highlighted a direct link between obesity‐related metabolic dysfunction and the development of adverse pulmonary vascular remodeling leading to PH, mediated by aberrant energy utilization across multiple organ systems (Kelley, 2005; Koves et al., 2008; Paulin & Michelakis, 2014; Wan et al., 2020). In our study, we leveraged a unique multi‐site blood sampling approach to discern metabolite differences across the SVC and PA, which may reflect diverse splanchnic tributaries draining into the IVC, to inform potential metabolic pathways operant in obesity‐associated PH. Previous multi‐site sampling studies focused on PAH and demonstrated significant trans‐RV gradients in dicarboxylic acids and acylcarnitines, which in turn were associated with measures of RV function and pulmonary vascular resistance (Chouvarine et al., 2020; Lewis et al., 2016). We now build upon these prior studies and leverage multi‐site metabolomic profiling to evaluate trans‐RV metabolite gradients in a sample with obesity and dyspnea, and examine associations with PH in this more general sample.

We found numerous metabolites that displayed gradients across the RV, including acylcarnitines, TCA cycle intermediates (i.e., succinic acid and pyruvate), nucleotide derivatives (guanine, orotic acid, and xanthine), and amino acids (alanine, pyroglutamic acid, and glutamic acid). Among the top metabolites that displayed higher concentrations in PA vs. SVC samples (implying potential contributions from IVC tributaries) were 3‐hydroxybutyric acid, known to be synthesized in the liver, and glutamic acid, a non‐essential amino acid known to potentiate adverse cardiometabolic effects and upregulate gluconeogenesis (Cheng et al., 2012; Maltais‐Payette et al., 2018). By contrast, one of the main metabolite classes associated with negative trans‐RV gradients were acylcarnitines, including CAR 5:0, CAR 8:0, and CAR 10:1. These have previously been linked to aberrant mitochondrial fatty acid oxidation in prior studies in obesity and cardiometabolic syndrome (Dambrova et al., 2022; Gao et al., 2016; Mihalik et al., 2010). Our findings add further granularity to acylcarnitine changes and association with cardiometabolic traits: in addition to demonstrating ∆MET in specific acylcarnitines, we also show that ∆MET in CAR 8:0 and CAR 18:0 are associated with HOMA‐IR, and CAR 20:4 is associated with HbA1c. Finally, we found that TCA cycle intermediates such as succinic acid and pyruvate displayed negative trans‐RV gradients in obesity (lower concentrations in the PA vs. SVC samples). Succinate is released in response to metabolic stress and has been shown to play a pro‐inflammatory role in obesity via the succinate receptor 1 (SUCNR1) (van Diepen et al., 2017). Similarly, pyruvate has been shown to be elevated in obese states, suggesting limited TCA cycle capacity with decreased conversion of pyruvate to acetyl CoA (Butte et al., 2015). Interestingly, in our sample, trans‐RV pyruvate gradients were positively associated with BMI and HOMA‐IR. Notably, pyruvate and other metabolites may play intermediary roles in multiple metabolic pathways. Herein, we highlight pertinent possible pathways with supporting literature, and future studies are warranted to further delineate metabolite functionality.

Beyond delineating ∆MET and associations with metabolic traits, we also identified their associations with PH. Specifically, among 22 trans‐RV metabolite gradients associated with PH, orotic acid had lower concentrations in PA versus SVC and was associated with greater odds of PH. In rat models of PH, orotate levels strongly correlated with hemodynamic indices of RV function and were suggested to play a protective role against vasoconstriction and hypertrophy (Hautbergue et al., 2021). We also found that trans‐RV uric acid concentrations were associated with PH. Prior data suggest that uric acid, produced from purine degradation, contributes to oxidative stress, inflammation, and endothelial damage that promotes progression of obesity and metabolic syndrome (Xiong et al., 2019). Lastly, primary bile acids (taurodeoxycholic, taurocholic, and glycocholic acids) were associated with greater odds of PH. Alterations in bile acids have been previously implicated in the pathogenesis of PAH through pro‐inflammatory gut dysbiosis and dysregulated synthesis within pulmonary vascular endothelial cells (Chouvarine et al., 2020; Moutsoglou et al., 2023; Zhao et al., 2014). Our findings extend prior studies focused on PAH, wherein ∆MET in dicarboxylic acids and acylcarnitines were associated with measures of RV function and pulmonary vascular resistance and demonstrate specific ∆MET operant across a wider spectrum of PH in the setting of obesity (Chouvarine et al., 2020).

Several limitations should be noted. We focused our sample on individuals with obesity and dyspnea, and thus generalizability to broader samples remains to be determined. Our study sample size had limited power to detect more modest effect sizes, including differences between cardiometabolic or PH subtypes. Because of the invasive nature of the study, we did not include healthy controls, and we acknowledge this limits any conclusions that can be extrapolated to non‐obese metabolically healthy individuals. Next, we used a targeted platform to analyze specific metabolites, and broader metabolic abnormalities may not be fully captured. We recognize that circulating metabolites are not organ or tissue specific, and cannot be definitively attributed to specific sources, nor can causal inferences be drawn. Due to the modest sample size, analyses examining correlations of trans‐RV metabolite gradients with cardiometabolic traits were viewed as exploratory. Finally, due to the observational study design, causality cannot be inferred between implicated metabolites and disease states.

In conclusion, we used multi‐site sampling to delineate trans‐RV metabolite gradients among individuals with obesity. We highlight positive trans‐RV metabolite gradients associated with cardiometabolic traits and/or PH, including BCAAs such as glutamic acid. Simultaneously, we identified multiple negative trans‐RV metabolite gradients associated with cardiometabolic traits and/or PH, including orotic acid, pyruvic acid, bile salts, and acylcarnitines. Taken together, these findings highlight potential molecular pathways that may underlie cardiometabolic disease and obesity‐driven PH. Further studies are warranted to elucidate the complex metabolic interplay between obesity, metabolic disease, and PH.

## FUNDING INFORMATION

JEH was supported by NIH grants R01‐HL134893, R01‐HL160003, R01‐HL168889, and K24‐HL153669. JMR was supported by NIH grants K23‐HL150327. LBK was supported by the National Institutes of Health (NIH) T32‐HL160522. ESL is supported by grants from the National Institutes of Health (K23‐HL159243), the American Heart Association (853922), and the Massachusetts Life Sciences Center.

## CONFLICT OF INTEREST STATEMENT

The authors declare no conflicts of interest.

## Supporting information


**Figure S1.** Metabolites with significant trans‐right ventricle gradients.

**Figure S2.** Correlation of significant Trans‐RV metabolite gradients with cardiometabolic traits. Correlation of 56 metabolites displaying significant trans‐RV gradients with cardiometabolic traits. Rows indicate metabolites with at least one significant cardiometabolic correlate (*p* <0.05). Heat map displays partial correlation coefficients of trans‐RV metabolic gradients with cardiometabolic traits (BMI, waist circumference, hemoglobin A1c, LDL cholesterol, and TGs) after adjusting for age and sex. Positive correlation coefficients are shown in red while negative coefficients are shown in blue (colors scaled between minimum value of −1 and maximum of 1). Absolute trans‐RV gradients are displayed in the right most column, with color scaling indicating directionality.

**Table S1.** Clinical and hemodynamic characteristics of overall sample and stratified by pulmonary hypertension status.

**Table S2.** Trans‐right ventricle gradients of all 379 profiled metabolites.

**Table S3.** Partial correlations of metabolites with clinical correlates, adjusted for age and sex.

**Table S4.** Associations between metabolite gradients and pulmonary hypertension.

## Data Availability

Data supporting the findings of this study are available from the corresponding author on reasonable request.
