# Supplementary figures and images for: Trans‐right ventricle metabolite gradients in obesity highlight multiple metabolic pathways
Source: Physiol Rep. 2025 May 11;13(9):e70323. doi: 10.14814/phy2.70323 (PMC12066817; doi:10.14814/phy2.70323)

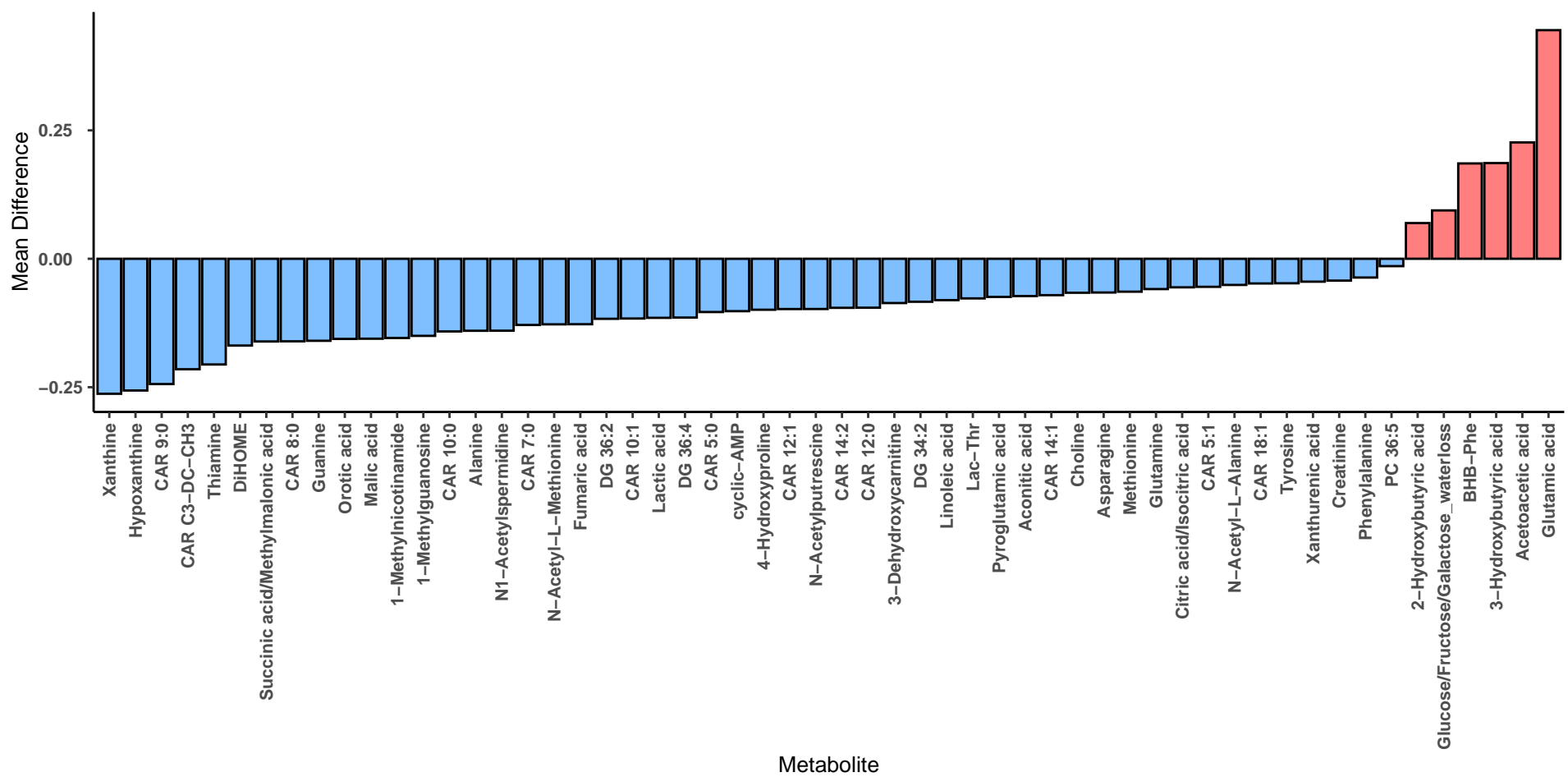

Supplement: Supplementary file 1 — Figure S1. Metabolites with significant trans‐right ventricle gradients. [file PHY2-13-e70323-s004.pdf]

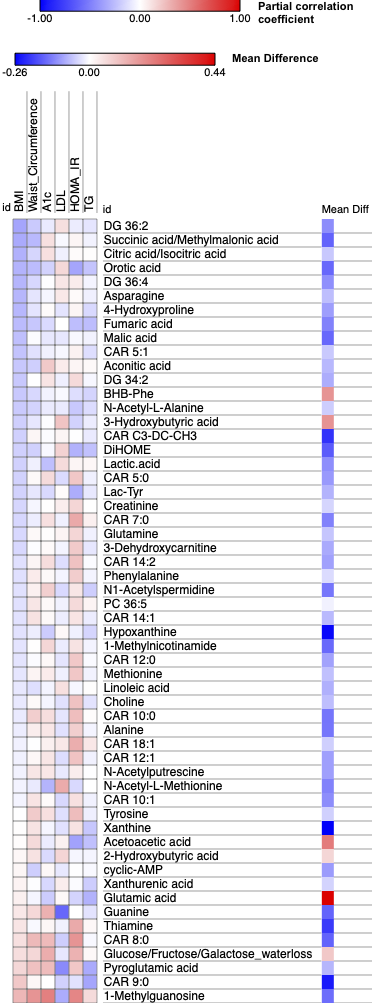

Supplement: Supplementary file 2 — Figure S2. Correlation of significant Trans‐RV metabolite gradients with cardiometabolic traits. Correlation of 56 metabolites displaying significant trans‐RV gradients with cardiometabolic traits. Rows indicate metabolites with at least one significant cardiometabolic correlate (p <0.05). Heat map displays partial correlation coefficients of trans‐RV metabolic gradients with cardiometabolic traits (BMI, waist circumference, hemoglobin A1c, LDL cholesterol, and TGs) after adjusting for age and sex. Positive correlation coefficients are shown in red while negative coefficients are shown in blue (colors scaled between minimum value of −1 and maximum of 1). Absolute trans‐RV gradients are displayed in the right most column, with color scaling indicating directionality. [file PHY2-13-e70323-s001.tif]
