# Supplementary material for: Trans‐right ventricle metabolite gradients in obesity highlight multiple metabolic pathways
Source: Physiol Rep. 2025 May 11;13(9):e70323. doi: 10.14814/phy2.70323 (PMC12066817; doi:10.14814/phy2.70323)
Supplement: Supplementary file 3 — Table S1. Clinical and hemodynamic characteristics of overall sample and stratified by pulmonary hypertension status. [file PHY2-13-e70323-s005.docx]

**Table 1. Clinical and Hemodynamic Characteristics of Overall Sample and Stratified by Pulmonary Hypertension Status.**

|  | **Total Sample (n=38)** | **PH**  **(n=18)** | **No PH**  **(n=20)** | **p-value** |
| --- | --- | --- | --- | --- |
| **Clinical characteristics** | | | | |
| Age, years | 57.9 [12.2] | 55.9 [10.9] | 59.7 [13.2] | 0.34 |
| Women, n (%) | 26 (68.4) | 12 (66.7) | 14 (70.0) | 0.83 |
| Caucasian, n (%) | 29 (76.3) | 12 (66.7) | 17 (85.0) | 0.18 |
| BMI, kg/m^2^ | 36.6 (5.5) | 38.6 [4.8] | 34.7 [5.6] | 0.03 |
| Waist circumference, cm | 115.5 [13.8] | 118.9 [11.7] | 112.4 [15.1] | 0.15 |
| HbA1c, % | 5.6 [0.5] | 5.7 [0.6] | 5.5 [0.4] | 0.18 |
| HDL cholesterol, mg/dL | 47 [13] | 50 [14] | 45 [12] | 0.25 |
| LDL cholesterol, mg/dL | 104 [34] | 106 [37] | 103 [32] | 0.81 |
| HTN history, n (%) | 16 (42.1) | 8 (44.4) | 8 (40.0) | 0.78 |
| Smoking history, n (%) | 16 (42.1) | 3 (16.7) | 13 (65.0) | <0.0001 |
| Diabetes, n (%) | 2 (5.3) | 1 (5.6) | 1 (5.0) | 0.94 |
| COPD, n (%) | 5 (13.2) | 1 (5.6) | 4 (20.0) | 0.19 |
| Statin use, n (%) | 14 (36.8) | 5 (27.8) | 9 (45.0) | 0.27 |
| Creatinine, mg/dL | 0.9 [0.2] | 0.8 [0.1] | 0.9 [0.2] | 0.30 |
| **Hemodynamic measures** | | | |  |
| mPAP, mmHg | 21.2 [6.4] | 26.8 [4.1] | 16.1 [2.9] | <0.0001 |
| PVR, WU | 1.6 [0.7] | 1.5 [0.7] | 1.6 [0.7] | 0.74 |
| PCWP, mmHg | 12.9 [5.7] | 17.1 [4.9] | 9.1 [3.2] | <0.0001 |
| RAP, mmHg | 7.9 [4.0] | 10.8 [3.2] | 5.2 [2.5] | <0.0001 |
| CO, L/min | 5.6 [1.4] | 6.1 [1.7] | 5.2 [1.1] | 0.06 |

Values are mean [standard deviation] unless otherwise denoted. BMI = Body mass index. CO = Cardiac output. COPD = Chronic obstructive pulmonary disease. HbA1c = glycated hemoglobin. HDL = high-density lipoprotein. HTN = Hypertension. LDL = Low-density lipoprotein. L/min = Liters per minute. mPAP = Mean pulmonary artery pressure. PCWP = Pulmonary capillary wedge pressure. PH = Pulmonary hypertension. RAP = right atrial pressure. PVR = Pulmonary vascular resistance. WU = Wood units.
